# New Medical Colleges

**Published:** 1881-12

**Authors:** E. J. Doering

**Affiliations:** Chicago, Ill.


### Domestic Correspondence.

### ARTICLE X.

EDITORS CHICAGO MEDICAL JOURNAL AND EXAMINER: — No one will dispute the assertion that there cannot be found a more enterprising and progressive city than Chicago; and it is therefore to me a matter of pleasure as well as pride that I am able to communicate to you and your readers a new venture, which cannot fail to add to the fame of this beautiful city. Recognizing the fact that the most advanced stage of civilization is inconsistent with a limited number of physicians and few professors of medicine, a number of gentlemen prominent in the profession in this city have taken the necessary steps to incorporate a new college, to be known as the Illinois State University of Medicine, to be located, of course, in Chicago. It is true that a similar institution, called the Chicago College of Physicians and Surgeons, is in process of organization, in order to meet "a want long felt," but the University of Medicine presents features so entirely distinct from the former, that they ought not to be confounded by any person possessed of average intelligence.

As is observed from the advertisements for professors in the various medical journals, the number of professorships in the College of Physicians and Surgeons will not exceed twenty, and the resident of any State in the Union is eligible for a professorship, if he possesses the necessary qualifications, which may be ascertained by addressing the Secretary (in confidence). Not so, however, in the Illinois State University of Medicine. In conversation with the accomplished Dean and other members of the prospective faculty, I learn that no one shall be eligible for a professorship unless he be a citizen of the State of Illinois, the incorporators being well aware that there is no need to go outside the State, or this city even, to secure abundant material for professorships.

Again, it is the determination of the incorporators to greatly enlarge the miserably small percentage of physicians who are professors, and by every means in their power to contribute to the very essence of medical progress by so enlarging the number of professorships in the university, that the percentage of physicians in this city, at least, who are not professors, will be very small indeed. To accomplish this laudable object, the number of professorships in the university will be unlimited, new chairs to be constantly created as additional candidates may appear. Such neglected branches of medicine and general science as the diseases of the umbilicus, ante-natal impressions, medical astrology, etc., will be duly represented by separate and distinct chairs.

I am also informed by the Dean that all graduates who are residents of this State will be presented with a lectureship in the summer course of the university, and the degree of Master of Arts. Many other features of interest which will distinguish the university might be mentioned, but as the prospectus will shortly be issued, I forbear to trespass any further on your limited space.

With the Illinois State University of Medicine, the Chicago College of Physicians and Surgeons, the Chicago Medical College, the Rush Medical College, the Woman's Medical College, the Hahnemann Medical College, the Chicago Homœopathic College and the Bennett Eclectic Medical College, all located in Chicago, we may indeed pride ourselves that we not only lead the United States, but all creation, in the matter of medical education, and assure ourselves that the time is not far distant which will truly be the millenium, when every citizen of Illinois will be a Doctor of Medicine, and every practitioner of the noble at a professor as well.

CHICAGO, ILL. E. J. DOERING.

